# Lipid spirals in *Bacillus subtilis* and their role in cell division

**DOI:** 10.1111/j.1365-2958.2008.06236.x

**Published:** 2008-06

**Authors:** Imrich Barák, Katarína Muchová, Anthony J Wilkinson, Peter J O'Toole, Nada Pavlendová

**Affiliations:** 1 Institute of Molecular Biology, Slovak Academy of Sciences 845 51 Bratislava 45, Slovakia; 2 Structural Biology Laboratory, Department of Chemistry, University of York York YO10 5YW, UK; 3 Technology Facility, Department of Biology, University of York York YO10 5YW, UK

## Abstract

The fluid mosaic model of membrane structure has been revised in recent years as it has become evident that domains of different lipid composition are present in eukaryotic and prokaryotic cells. Using membrane binding fluorescent dyes, we demonstrate the presence of lipid spirals extending along the long axis of cells of the rod-shaped bacterium *Bacillus subtilis*. These spiral structures are absent from cells in which the synthesis of phosphatidylglycerol is disrupted, suggesting an enrichment in anionic phospholipids. Green fluorescent protein fusions of the cell division protein MinD also form spiral structures and these were shown by fluorescence resonance energy transfer to be coincident with the lipid spirals. These data indicate a higher level of membrane lipid organization than previously observed and a primary role for lipid spirals in determining the site of cell division in bacterial cells.

## Introduction

Cells normally divide by a process of binary fission in which a septum partitions the cell with high precision into two equal halves. The survival of the cell line depends on the equal partitioning of the chromosomal and cytoplasmic material into the two daughter cells. How cells define the site of cell division with such high accuracy is therefore a question of great interest and importance. In rod-shaped bacteria, such as *Escherichia coli* and *Bacillus subtilis*, a defining event in cell division is the polymerization at the site of septation of the tubulin-like protein FtsZ into a ring, called the Z-ring (Rothfield *et al*., 2005). Two distinct mechanisms contribute to accurate placement of the Z-ring and the entire division machinery: the Min system (Rothfield *et al*., 2005) and nucleoid occlusion (Woldringh *et al*., 1990).

The action of the Min system proteins prevents Z-ring formation and septation at unwanted polar sites so that by default, the septum forms at mid-cell. The MinCDE proteins in dividing *E. coli* interact with one another and with the cell membrane to generate a concentration gradient of the cell division inhibitor MinC (Hu and Lutkenhaus, 1999; Raskin and de Boer, 1999). This involves oscillation of the Min proteins along a spiral trajectory from pole to pole, the result of which is a MinC concentration minimum at mid-cell (Fu *et al*., 2001). The dynamic properties of the system are conferred by MinE, which plays a topological role, and MinD an ATPase that uses an amphipathic positively charged C-terminal α-helix to associate reversibly with the cytoplasmic membrane (Szeto *et al*., 2002; Hu and Lutkenhaus, 2003). This membrane targeting sequence (MTS) is conserved in all MinD homologues. The MTS of *E. coli* has been shown to bind preferentially to anionic phospholipids and several of the hydrophobic residues within this sequence insert into the cell membrane lipid bilayer (Mileykovskaya *et al*., 2003). The MTS of *B. subtilis* MinD is three amino acid residues longer than the MTS of *E. coli* MinD, indicating a possible higher affinity for the membrane (Szeto *et al*., 2002).

Although the Min proteins are widely conserved, they are not present in all bacteria. *B. subtilis*, like *E. coli*, has MinC and MinD homologues, which are important for the prevention of asymmetric septation during vegetative growth. However, *B. subtilis* lacks MinE and an unrelated protein DivIVA is responsible for spatial regulation of MinCD (Marston *et al*., 1998; Marston and Errington, 1999). DivIVA has no sequence similarity to MinE, it has a different quaternary structure (Zhang *et al*., 1998; Stahlberg *et al*., 2004) and it clearly functions differently to MinE. DivIVA is stably associated with the cell poles, to which it recruits MinCD, probably by direct interaction with MinD (Marston *et al*., 1998; El Karoui and Errington, 2001). The Min proteins do not oscillate in *B. subtilis*; instead, they localize to, and emanate from, the poles of the cell, creating a concentration gradient of the cell division inhibitor, MinC, with the concentration minimum occurring at mid-cell (Marston *et al*., 1998; Marston and Errington, 1999). Although, the action of this Min system appears to be static at the cellular level, it is likely that its organization is dynamic at the molecular level. One puzzle regarding the Min system is the observation of Min protein spirals in *E. coli* but not in *B. subtilis*. Little is known, however, of the origin of these spiral structures. Here we report the discovery of lipid spirals in *B. subtilis*, and their colocalization with spirals of GFP–MinD. We present evidence that the lipid spirals are enriched in anionic phospholipids, suggesting a mechanism for membrane binding by MinD based on complementary charge.

## Results and discussion

### Lipid spiral-like structures in *B. subtilis* cells

**Fig. 1:**
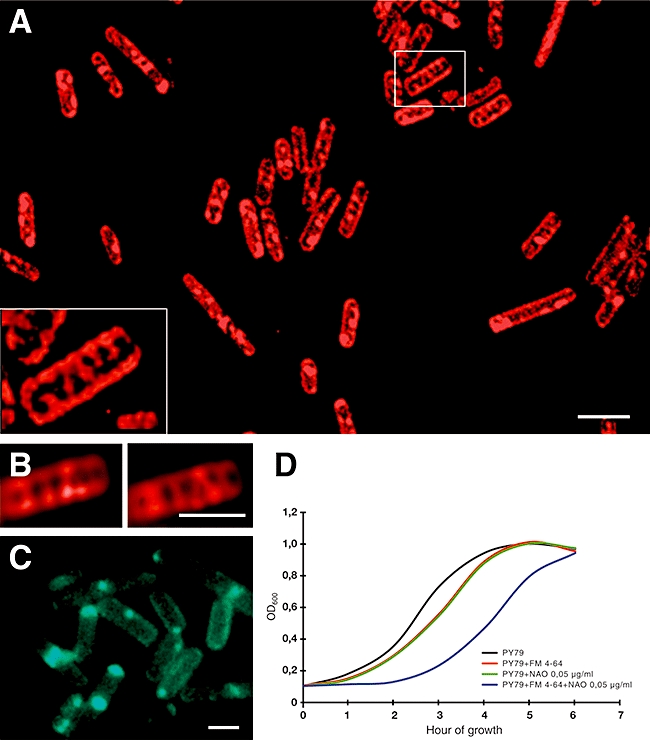
Lipid spirals in *B. subtilis*. A. Visualization of FM 4-64 (red) signals in wild-type *B. subtilis*. The lipid helices were discerned by assembling a series of Z-stack images taken in successive planes followed by sharpening by deconvolution (inset images). B. FM 4-64 signals in two different focal planes of a deconvolved cell. C. Staining of wild-type *B. subtilis* with NAO to visualize cardiolipin domains at the poles and at the site of septation. D. Growth curves of wild-type *B. subtilis* grown in DSM media without supplement (black), supplemented with FM 4-64 (red), NAO (green) and their combination (blue). Each growth curve represents the average of data from three independent experiments. The graph shows the evident negative effect on cell growth when both dyes are present. Scale bar for A is 2 μm and for B and C, 1 μm.

The membrane structure of wild-type *B. subtilis* cells was examined by fluorescence and confocal microscopy using the fluorescent probes FM 4-64, FM 1-43 and FM 5-95. After growth of cells to OD_600_ = 0.3 in Difco sporulation medium (DSM) at 37°C (see *Experimental procedures*), we observed a helical or spiral distribution of the fluorescent dyes (Fig. 1A and B). Helices were seen in all cells inspected although, in some cells, they were less well-resolved and appeared more like the lipid domains which have been described previously (Matsumoto *et al*., 2006). Interestingly, the lipid spirals persisted throughout vegetative growth and into stationary phase. These structures appeared to consist of intertwining ribbons many of which were prominent near the poles. Most of the filaments were helical along their entire length (Fig. 1A and B). Although, the best-resolved lipid helices were observed with FM 4-64, the FM 1-43 and FM 5-95 dyes exhibited a similar pattern of spiral localization. These helical lipid structures have not been described previously, an omission that may be explained by the advanced instrumentation used here which generates lower background fluorescence and improved resolution in the fluorescent and confocal images. In the light of these observations, we re-examined the localization of the FM dyes in *E. coli* cells. As reported previously (Fishov and Woldringh, 1999), we observed bands or dots of FM 4-64 staining but no discernible spiral structures (not shown), implying either that lipid spirals are not present in this organism or that the additional outer membrane layer surrounding the cell obscures their presence.

Time-lapse fluorescence microscopy revealed that the lipid spirals in *B. subtilis* persist for several minutes until the FM 4-64 dye becomes bleached out (data not shown). Furthermore, after labelling with FM 4-64 and bleaching of a region of interest (ROI), we observed no fluorescence recovery after photobleaching (FRAP) in the ROI on membranes of living cells over a period of 10 min (not shown). In contrast, when FM dyes were added to the media during the FRAP experiments, the bleached ROI partially recovered fluorescence on a timescale of 10–30 s. These observations indicate that dye molecules from neighbouring helical regions have little or no mobility, or that a mechanical obstruction prevents their diffusion into the bleached region.

One explanation for the failure of the FM dye to diffuse into the bleached region is interaction of the lipid with a specific membrane-associated protein. One candidate, MinD, which colocalizes with the lipid spirals (see below), can be excluded because FM 4-64 spiral-like signals were also observed in strain IB1056 where no MinD is present. An alternative possibility is that binding of the fluorescent dye to the lipid structure induces a rigidity which prevents lateral diffusion/exchange of lipid molecules. However, neither FM 4-64 nor another membrane dye 10-*N* nonyl-acridine orange, NAO (see below), has been reported to have a detectable influence on cell growth or other cellular processes examined (Pogliano *et al*., 1999; Kawai *et al*., 2004). In the light of our FM 4-64 FRAP experiments, we re-examined the influence of each of the dyes on cell growth. As seen in Fig. 1D, these dyes had only small effects on the cell growth when used alone but, when used together, there was a clear slow-down in the cell growth rate (Fig. 1D). We infer that this growth retardation may be caused by the rigidity induced by the dye(s) in the lipid spirals.

### MinD–GFP localizes as spirals in *B. subtilis* cells

It has been proposed that heterogeneity in the distribution of membrane phospholipids contributes to division site selection in bacteria by modulating the interactions of MinD with the membrane (Mileykovskaya and Dowhan, 2005). In the light of the lipid spiral structures observed above, we re-investigated MinD localization in *B. subtilis*, using xylose-inducible *gfp–minD* fusions chromosomally inserted at the *amyE* locus. We prepared strain IB1060 which retains the wild-type *minD* gene and strain IB1059 in which *minD* is disrupted (Table 1). By growing cells of strain IB1059 in the presence of different concentrations of xylose, we were able to show, using immunodetection methods, that the levels of GFP–MinD produced in the cells could be controlled (data not shown). The cells became elongated if the xylose concentration exceeded 0.1% as a result of overproduction of GFP–MinD and inhibition of mid-cell division. In contrast, cells grown in the absence of xylose had the expected mini-cell phenotype associated with loss of Min system inhibition of division at the cell poles.

**Table 1:** Strains and plasmids.

Strain	Genotype	Source/reference
*E. coli*		
MM294	*endA1 hsdR17 supE44 thi-1 recA*^+^	Backman *et al*. (1976)
*B. subtilis*		
PY79	Prototroph	Youngman *et al*. (1984)
MO1099	*amyE::erm MLS pheA1 trpC2*	Guerout-Fleury *et al*. (1996)
IB1056	MO1099 Δ*minD::Cm*	This work
IB1059	IB1056 *amyE::Pxyl-gfpmut1-minD spc*	This work
IB1060	MO1099 *amyE::Pxyl-gfpmut1-minD spc*	This work
BFA2809	*trpC2 pgsA::pMutin4*	Kobayashi *et al*. (2003)
IB1061	BFA2809 *amyE::Pxyl-gfpmut1-minD spc*	This work
IB1062	MO1099 *amyE::Pxyl-minD spc*	This work
IB1063	IB1056 *amyE::Pxyl-gfpmut1-minD*Δ*3 spc cam*	This work
IB1064	IB1056 *amyE::Pxyl-gfpmut1-minD*Δ*4 spc cam*	This work
IB1065	IB1056 *amyE::Pxyl-gfpmut1-minD*Δ*6 spc cam*	This work
IB1066	IB1056 *amyE::Pxyl-gfpmut1-minD*Δ*15 spc cam*	This work
IB1067	IB1056 *amyE::Pxyl-gfpmut1-minD*Δ*24 spc cam*	This work
IB1073	MO1099 *amyE::Pxyl-gfpmut1 spc*	This work
Plasmids		
pSG1151	*bla cat gfpmut1*	Lewis and Marston (1999)
pSG1154	*bla amyE3′ spc Pxyl-gfpmut1 amyE5′*	Lewis and Marston (1999)
pSG1729	*bla amyE3′ spc Pxyl-gfpmut1 amyE5′*	Lewis and Marston (1999)
pSGminDdel	*bla cat*Δ*minD*	This work
pSGminD	*bla amyE3′ spc Pxyl-gfpmut1-minD amyE5′*	This work
pSGminD2	*bla amyE3′ spc Pxyl-minD amyE5′*	This work
pSGminDΔ3	*bla amyE3′ spc Pxyl-gfpmut1-minD*Δ3 *amyE5′*	This work
pSGminDΔ4	*bla amyE3′ spc Pxyl-gfpmut1-minD*Δ4 *amyE5′*	This work
pSGminDΔ6	*bla amyE3′ spc Pxyl-gfpmut1-minD*Δ6 *amyE5′*	This work
pSGminDΔ15	*bla amyE3′ spc Pxyl-gfpmut1-minD*Δ15 *amyE5′*	This work
pSGminDΔ24	*bla amyE3′ spc Pxyl-gfpmut1-minD*Δ24 *amyE5′*	This work

**Fig. 2:**
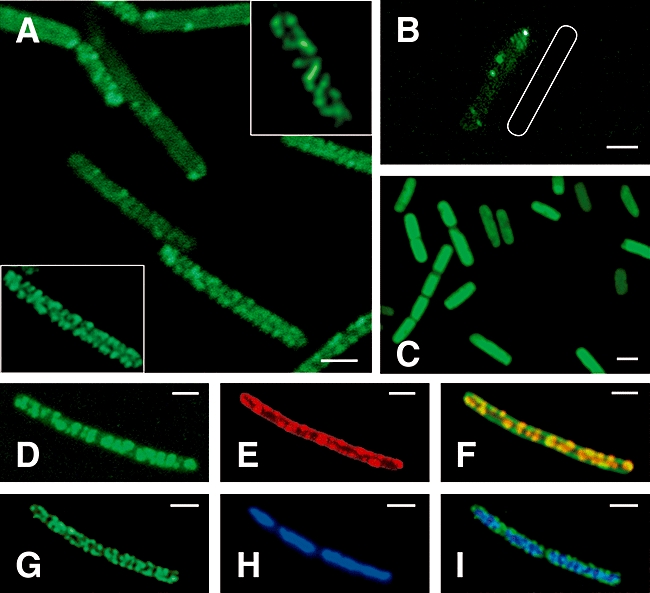
MinD spirals in *B. subtilis*. Visualization of GFP–MinD (green) signals in strain IB1060 (A) and MinD immunofluorescence (green) signals in strain IB1062 (B) by fluorescence microscopy. A shows the localization of GFP fused to MinD in strain IB1060 after *gfp-minD* induction with 0.1% xylose while C shows the GFP (green) signal in the control strain IB1073, following similar induction. B shows imunnofluorescence analysis of MinD localization in strain IB1062 after *minD* induction with 0.05% xylose where MinD is localized preferentially at the cell poles or at the site of septation, with a weak fluorescence signal radiating out from one of the poles. The shape of the fluorescently imaged cell is drawn next to it as a white outline. Vegetative cells in all cases were grown to mid-exponential phase in DSM medium. D–F show confocal images of strain IB1060 after *gfp–minD* induction with 0.1% xylose; these are visualizations of GFP–MinD (D), FM 4-64 (E), and a rendered image of the overlay of the images in D and E (F). The brightness of the GFP–MinD spirals in IB1060 could be altered by varying the level of *gfp–minD* induction, suggesting that the spiral structures can accommodate varying amounts of protein. The GFP–MinD helices were discerned by assembling a series of Z-stack images taken in successive planes followed by sharpening by deconvolution (inset images in A) and rendering in the case of F. G and H are fluorescence microscopy visualizations of GFP–MinD and nucleoids stained with DAPI, respectively, in strain IB1060 after *gfp–minD* induction with 0.05% xylose. I is a merged image of G and H. Polar localization of GFP–MinD and GFP–MinD spirals surrounding the nucleoids are evident. Scale bars for all panels represent 1 μm.

We observed localization of GFP–MinD at the cell poles as observed previously (Marston and Errington, 1999). Strikingly, we observed further localization of GFP–MinD as spirals in both strains IB1060 (Fig. 2A, D and G) and IB1059 (data not shown). In the control strain IB1073 expressing free GFP, the fluorescence signal is dispersed throughout the cytoplasm (Fig. 2C). In strain IB1060, spirals could be discerned even at the lowest xylose concentration used (0.05%) when the intracellular GFP–MinD concentration resembles that of MinD in wild-type cells as determined by Western blot analysis (not shown). MinD spiral formation was also tested by immunofluorescence microscopy in strain IB1062 (Table 1) using a polyclonal anti-MinD antibody (see *Experimental procedures*). When *minD* expression in IB1062 was induced by low concentrations of xylose (0.05%), immunofluorescence microscopy showed MinD signals preferentially localized at or near the cell poles. In some of these cells, it was also possible to observe hazy, possibly spiral-like signals radiating out from one of the poles (Fig. 2B). Similar spiral structures to those seen with the GFP–MinD construct were observed when *minD* expression was induced by 0.1% xylose or higher. However, these spirals were not as well-resolved as those observed by the GFP–MinD fluorescence, perhaps because of non-specific binding by the polyclonal anti-MinD antibody (not shown).

**Fig. 3:**
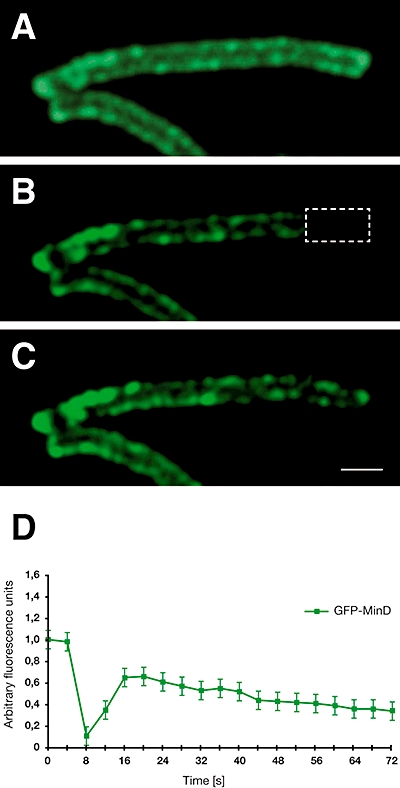
FRAP analysis of MinD spirals. FRAP analysis of GFP–MinD in IB1060 after *gfp–minD* induction with 0.1% xylose. The marked segment of the cell was photobleached by exposure to 488 nm laser light and subsequently photographed every 4 s for several minutes. The cell is shown before the bleach (A), immediately after bleaching (B) and 12 s after bleaching (C). Graph D shows the change in intensity of GFP signals during the FRAP experiment as a function of time in seconds. Photo bleaching was initiated after *t* = 4 s. Scale bar for A–C is 1 μm.

Pole-to-pole oscillation of the Min system proteins occurs in *E. coli* (Hu and Lutkenhaus, 1999; Raskin and de Boer, 1999) but not *B. subtilis* and, indeed, we observed no change in the localization of GFP–MinD fluorescence in IB1060 cells monitored by time-lapse microscopy over 5 min following xylose (0.1%) induction. Next, we monitored GFP–MinD diffusion in IB1060 cells by FRAP. Following photobleaching of defined areas of the cell, fluorescence was recovered within 12 s in all of the living cells tested (Fig. 3A–D). Similar observations were made with cells prepared in the presence of chloramphenicol, eliminating the possibility that newly synthesized GFP–MinD was being inserted into the bleached areas. In a further control experiment, we observed no recovery of fluorescence in the helical structures following photobleaching of whole cells. We conclude that recovery of fluorescence is due to proteins from adjacent helical regions entering the bleached zone either by: (i) diffusion along the helix or (ii) more appealingly, based on what is known of MinD in *E. coli*, by a membrane dissociation and re-association process (Drew *et al*., 2005).

### MinD spirals and lipid spirals colocalize in the cell membrane

**Fig. 4:**
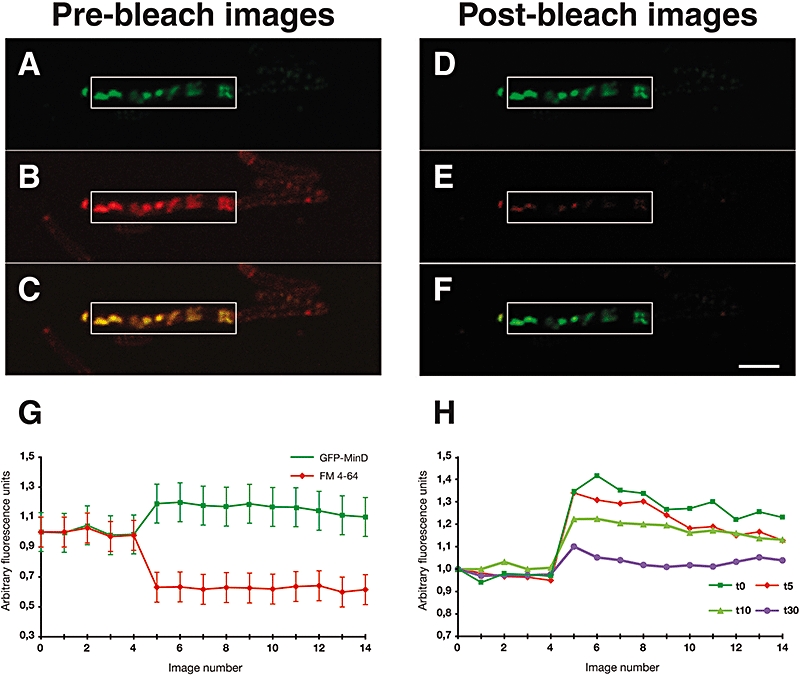
Colocalization of the lipid and MinD spirals. FRET between GFP–MinD and lipids labelled with FM 4-64. Images A and D show the GFP–MinD (donor) fluorescence signal before and after photobleaching of the acceptor FM 4-64. A clear increase in fluorescence intensity can be seen. Images B and E show the FM 4-64 (acceptor) fluorescence signal before and after photobleaching. A decrease in intensity can clearly be seen within the marked region. Images C and F represent the merged images A, B and D, E respectively. Graph G shows the mean change in intensity of both fluorophores during the image acquisition time. A bleaching of FM 4-64 coincident with an increase in GFP fluorescence intensity is apparent. Graph H shows the change in intensity of GFP signals during the FRET experiments as a function of time. The dark-green curve (squares) represents cells observed immediately after plating onto agarose-coated glass slides. The light-green curve (triangles) represents cells observed after 10 min, the red curve (diamonds) cells observed after 15 min and the purple curve (circles) cells observed after 30 min. Scale bar for images A–F is 2 μm.

To examine the spatial relationship between the fluorescent spirals produced by FM 4-64 and those produced by GFP–MinD, suitable images were overlaid (Fig. 2D–F). As shown in Fig. 2F, the fluorescent signals from GFP–MinD foci exhibit a high degree of correlation with those from FM 4-64. These signals were therefore examined using flourescence resonance energy transfer (FRET) (see *Experimental procedures*). Figure 4A–F shows a representative dual-stained image collected during FRET acceptor bleaching experiments with the rectangle representing a ROI bleached with a 561 nm laser. The acceptor FM 4-64 signal, representing the lipid structure (Fig. 4B, E and G), is reduced after photobleaching. By contrast, the intensity of the donor GFP–MinD signal inside the selected ROI increases following bleaching (Fig. 4A, D and G). This is emphasized in the merged images that show a reduction in the intensity of the red and an increase in that of the green signal within the selected ROI (Fig. 4C and F). The complete FRET data were generated on five separate occasions from 40 cells and 98 selected ROIs, and the mean FRET for selected areas inside the ROI was determined to be 21 ± 6.2%. The FRET acceptor bleaching data were validated by control experiments giving an overall FRET efficiency of 17 ± 6.2% (see *Experimental procedures*). This number is probably underestimated because of the fact that the FRET efficiency decreases with time as shown in Fig. 4H. The highest FRET efficiencies were more than 30% in cells observed immediately after plating onto agarose-coated glass slides (time 0 in Fig. 4H) and decreased to 8% after 30 min (Fig. 4H). Thus, only FRET data that were collected within 20 min of plating the cells onto slides were analysed.

The FRET experiments indicate that the FM 4-64 lipid dyes and the GFP moieties of GFP–MinD are in close proximity to one another (less than 10 nm apart and most probably much closer) in a high proportion of molecular pairs. This demonstrates their colocalization in the cell membrane, and therefore the close spatial interaction of the lipid and GFP–MinD spiral structures.

### Lipid organization and GFP–MinD localization in cells with altered lipid composition

**Fig. 5:**
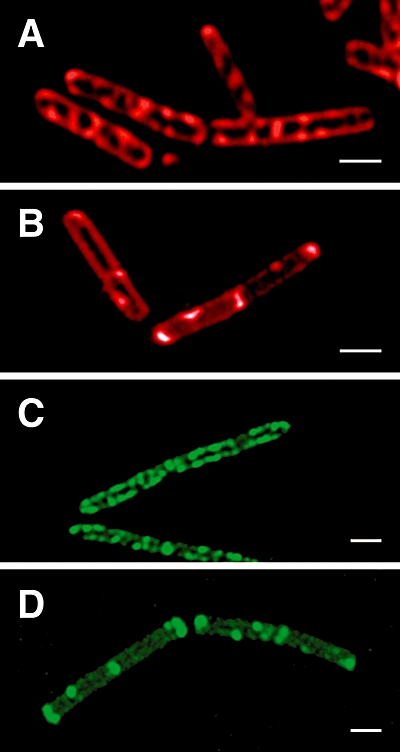
Dependence of lipid and GFP–MinD spiral formation on the presence of negatively charged lipids. A and B show FM 4-64 fluorescence signals for BFA2809 cells grown in the presence (A) or absence (B) of 100 μM IPTG. Images C and D are visualizations of GFP–MinD in IB1061 cells after *gfp–minD* induction with 0.1% xylose and growth in the presence (A) or absence (B) of 100 μM IPTG. Expression of *pgsA* from the IPTG-inducible promoter is required for the spiral localization of both the FM 4-64 dye and GFP–MinD (A and C). In the absence of IPTG (B and D), there is a redistribution of the FM 4-64 and GFP–MinD signals from the spirals (which are no longer evident) to the cell poles and to the sites of septation, where the relative fluorescence intensity increases. Scale bars for all images represent 1 μm.

All three FM dyes used in our experiments are cationic styryl compounds. Their net charge of +2 prevents their diffusion through membranes and they are not usually found in the cytoplasm (Brumback *et al*., 2004). The dyes are expected, therefore, to have a higher affinity for membranes enriched in negatively charged phospholipids. There are two major anionic lipids in the membranes of *B. subtilis* cells, phosphatidylglycerol (PG) and cardiolipin representing 40% and 25%, respectively, of the glycerophosphate-based lipid pool (Lopez *et al*., 1998; Fishov and Woldringh, 1999). This implies that the fluorescent spirals observed after treatment with the FM dyes define structures in the membrane that are richer relative to the bulk lipid in PG and cardiolipin. To probe the composition of the lipid spirals, we analysed cells in which the biosynthesis of PG phospholipids was perturbed. The *B. subtilis pgsA* gene encodes PG phosphate synthase, an essential enzyme in the biosynthesis of the anionic phospholipids, PG and cardiolipin (de Mendoza *et al*., 2002). The subcellular distribution of the FM dyes was analysed in *B. subtilis* BFA2809, a strain in which the expression of *pgsA* is under the control of the IPTG-inducible P_spac_ promoter (Kobayashi *et al*., 2003; Campo *et al*., 2004). Clear lipid spirals were discernible only in cells in which *pgsA* expression was induced (Fig. 5A). In cells where the expression of *pgsA* was not induced, the FM dyes were principally located on the periphery of the cells, at the cell poles and at the site of septation (Fig. 5B), and lipid spirals were either absent or not detectable. We conclude that the formation of lipid spirals is partly or wholly dependent on the presence in the membrane of anionic phospholipids. As all three fluorescent dyes have doubly charged cationic head groups, their preferential binding to membranes rich in negatively charged phospholipids may be explained by favourable electrostatic interactions.

To distinguish whether one or both of the anionic phospholipids is enriched in the spirals, we stained cells with the cardiolipin-specific dye NAO. As previously demonstrated (Kawai *et al*., 2004), the fluorescent dye was preferentially distributed in the septal regions and at the poles of *B. subtilis* cells (Fig. 1C). While cardiolipin may be present at much lower levels on the spirals, it is reasonable to assume that of the two major anionic phospholipids, PG and cardiolipin, PG is the principal component in the observed spirals.

Cells lacking both PG and cardiolipin are not viable. However, cells depleted of these components by removal of the *pgsA* inducer survive for several hours. This is because their function can be partially substituted by the anionic precursors, phosphatidic acid and CDP-diacylglycerol which accumulate in the absence of PG phosphate synthase (Mileykovskaya and Dowhan, 2005). These species are also recognized by the positively charged FM dyes (Fig. 5B).

To test the dependence of GFP–MinD spiral formation on the presence of negatively charged lipids, we prepared strain IB1061 (Table 1), a derivative of BFA2809 in which *gfp–minD* under the control of a xylose-inducible promoter has been inserted at the *amyE* locus. As for strain BFA2809, we examined cells grown in the presence or absence of 100 μM IPTG after induction of GFP–MinD expression with 0.1% xylose. In the absence of *pgsA* induction, GFP–MinD localized mainly at the poles of the cell, at the site of septation or as dots along the cell length (Fig. 5D). Clear spiral structures were not observed. Preferential polar occurrence of GFP–MinD in these cells can be explained by the presence of DivIVA which is a key determinant of MinCD complex accumulation at the poles and division sites in the wild-type *B. subtilis* (Edwards and Errington, 1997). Growth of strain IB1061 in the presence of IPTG (with expression of *pgsA*) produced cells with clearly discernible spirals of green fluorescence (Fig. 5C). The membrane phospholipid composition is therefore an important determinant of the spiral localization of the cell division regulator MinD. Interestingly, previous studies with liposomes have demonstrated that the extent of association of MinD with membranes is determined by their phospholipid composition, with a higher affinity exhibited towards anionic as opposed to zwitterionic phospholipids (Mileykovskaya *et al*., 2003).

### The C-terminal domain of MinD is crucial for spiral localization

**Fig. 6:**
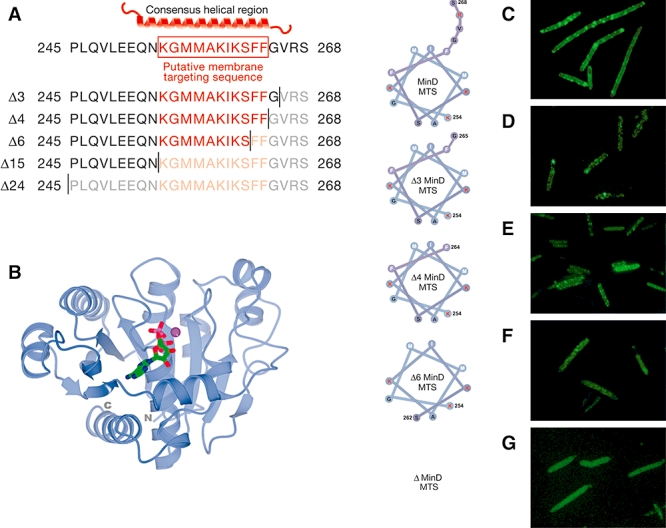
The MTS is required for the spiral localization of GFP–MinD. A. The C-terminal residues of MinD with the MTS motif shown in red. A putative α-helix is drawn above the sequence of the wild-type MinD. The C-terminal deletion mutants analysed in this work are indicated below. B. Ribbon drawing of the crystal structure of MinD from *Pyrococcus furiosus* (light-blue ribbon) with bound Mg-ADP (cylinder representation and coloured by element) (1G3Q) (Hayashi *et al*., 2001). The C-terminal region is poorly ordered (with eight residues missing) in this structure and in two other structures of MinD from thermophiles (Cordell and Lowe, 2001; Hayashi *et al*., 2001; Sakai *et al*., 2001), consistent with the notion that the helical structure forms only upon membrane binding. C–G. Helical-wheel representation of the putative MTS in wild-type MinD and the MinD deletion derivatives alongside fluorescence micrographs showing the localization in *B. subtilis* cells of the respective proteins fused to GFP. Hydrophobic residues are denoted by white letters and positively charged residues are shown by red letters. Residue Lys254 (K254) and the C-terminal residues of MinD and its deletion derivatives are numbered.

The sequence at the C-terminus of MinD which is conserved across archaea, eubacteria and chloroplasts has been shown to mediate interactions with membrane lipids *in vitro* and to be essential for membrane localization *in vivo* (Szeto *et al*., 2002). This MTS is not fully resolved in the three crystal structures of MinD homologues from hyperthermophilic archaebacteria (Fig. 6B) (Cordell and Lowe, 2001; Hayashi *et al*., 2001; Sakai *et al*., 2001). There is nevertheless good evidence to suggest that it forms, at least upon membrane binding, an amphipathic α-helix, one face of which is lipophilic while the other face is positively charged (Szeto *et al*., 2002). This amphipathic α-helix can align parallel to the membrane surface so that its hydrophobic face interacts with lipid acyl chains, while the cationic side-chains on the opposite face of the helix interact with the head groups of anionic phospholipids (Cornell and Taneva, 2006). Favourable electrostatic interactions may therefore explain the preference of MinD for membranes enriched in anionic phospholipids. We tested whether the predicted helical structure of the MTS is important for the specific membrane localization of MinD on a helical trajectory by examining the localization of several GFP–MinD truncation mutants (Fig. 6).

First, we examined the localization of GFP–MinDΔ24 in which the 24 C-terminal residues of the MinD moiety were removed. This mutant failed to exhibit a spiral pattern of membrane localization (Fig. 6G) with the fluorescence signal diffusely distributed throughout the cytoplasm. A 24 residue C-terminally truncated mutant of *E. coli* MinD was also found to reside in the cytoplasm (Szeto *et al*., 2002). A similar pattern of localization of the fluorescence signal was observed in IB1066 cells expressing GFP–MinDΔ15 (not shown). By contrast, the mutants GFP–MinDΔ3 and GFP–MinDΔ4, which lack three and four C-terminal residues, respectively, exhibited a spiral localization of the GFP signal identical to that of the full-length protein (Fig. 6C–E). Cells expressing MinDΔ6 exhibited an intermediate phenotype with some cells exhibiting a spiral localization of the fusion protein while others did not (Fig. 6F).

High-level expression of GFP–MinD induced by addition of > 0.1% xylose normally causes filamentation as a result of excessive cell division inhibition. In contrast, in isogenic strains overexpressing GFP–MinDΔ24 and GFP–MinDΔ15, filamentation was not observed (not shown). This implies that failure of MinD to localize as spirals correlates with loss/lowering of its function as a cell division inhibitor. Taken together, these results show that the C-terminal region of MinD, containing the putative MTS, is crucial for targeting the protein to the membrane on a spiral trajectory.

### Concluding remarks

**Fig. 7:**
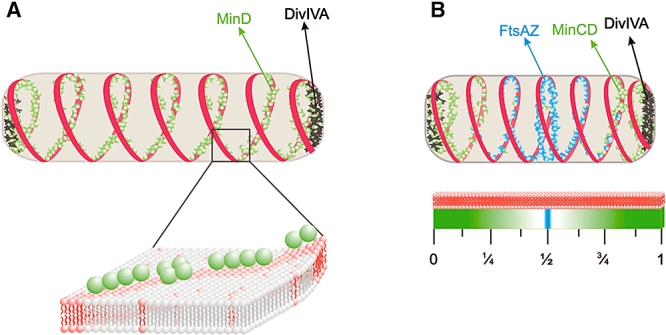
Role of lipid and MinD spirals in cell division. Lipid spirals (red strips) and MinD (green spheres) spirals are shown in cells (A) overexpressing MinD–GFP and (B) expressing wild-type levels of MinD. The polar-localizing protein DivIVA is represented as black dots. MinD/MinD–GFP associates preferentially and reversibly with anionic phospholipids, which are enriched in the spirals, and is attracted by DivIVA to the cell poles. MinD–GFP overexpression in A produces elongated cells with visible fluorescent protein spirals along the length of the cell. With wild-type levels of MinD (B), we propose that the concentration gradient across the cell is sufficiently sensitive to serve as a measuring device for the mid-cell plane where MinD (and therefore MinC) concentrations are at a minimum. FtsZ and FtsA (blue spheres) also localize on spiral-like structures (Peters *et al*., 2007), accumulating where the MinCD concentration is lowest, leading to Z-ring formation at the mid-cell.

The earliest apparent event in cell division in bacteria is the formation of an FtsZ ring (Z-ring) at the future septation site. During vegetative growth of *E. coli* and *B. subtilis*, the Z-ring forms at mid-cell where the cells subsequently divide. At least two distinct mechanisms contribute to placement of the division machinery: the Min system and nucleoid occlusion (Harry, 2001; Barák and Wilkinson, 2007). The idea that a concentration gradient in the Min system proteins serves as the measuring device for defining the mid-cell site of cell division is well established. In *E. coli*, this is achieved through the oscillation of the Min proteins from pole to pole (Hu and Lutkenhaus, 1999; Raskin and de Boer, 1999; Shih *et al*., 2003). In *B. subtilis*, different mechanisms are thought to operate because protein oscillation is not observed and DivIVA, which attracts MinCD to the cell poles, takes the place of MinE (Edwards and Errington, 1997). As most other rod-shaped bacteria have MinCDE or MinCD/DivIVA systems, it is generally assumed that they follow cell division site recognition strategies similar to those of *E. coli* or *B. subtilis*. The discovery here of lipid spirals recognized by MinD in *B. subtilis* allows the models for cell division site selection in the two systems to be brought closer together. Either through protein oscillation or through preferential attraction to the poles along an anionic helical track (Fig. 7), a longitudinal concentration gradient in MinD is formed which is capable of defining the mid-cell plane with high precision for accurate cell division.

It has been shown very recently that FtsZ and its membrane-tethering partner, FtsA, form dynamic helical structures in vegetatively growing *B. subtilis* cells (Peters *et al*., 2007). This complements the earlier discovery in sporulating cells of FtsZ structures that emanate from the Z-ring at mid-cell and migrate along a helical trajectory to polar sites of cell division (Ben-Yehuda and Losick, 2002). FtsA, like MinD, has an amphipathic positively charged α-helix that is required for localization to the membrane (Yim *et al*., 2000). Preferential binding to the spiral anionic phospholipid structures described here, therefore, provides an obvious and testable explanation for the spiral localization of FtzA and FtsZ. The lipid spirals described here may also provide a structure onto which components of the secretory apparatus attach. SecA, SecY and pre-AmyQ exhibit spiral patterns of localization, which in the case of SecA is dependent on the presence of acidic PG phospholipids (Campo *et al*., 2004). It is likely that structures other than the anionic lipid spirals described here also determine helical distributions of proteins. It is known, for example, that the cell shape maintenance protein MreB forms spiral structures (Daniel and Errington, 2003) although these are not coincident with those formed by MinD, at least in *E. coli* (Shih *et al*., 2005). Variability in structure and lipid composition may therefore be a general factor in shaping the interactions of membranes and proteins.

## Experimental procedures

### General methods

Growth of *B. subtilis* cell cultures, the preparation and transformation of competent cells, and the selection of transformants were performed as described previously (Harwood and Cutting, 1990). *B. subtilis* cells were grown at 37°C in liquid Luria–Bertani (LB) or DSM supplemented, when required, with antibiotics: chloramphenicol, 5 μg ml^−1^; erythromycin plus lincomycin, 1 and 25 μg ml^−1^ respectively; spectinomycin, 100 μg ml^−1^. For induction of the P_*xyl*_ -*gfpmut1-minD* and P_*xyl*_*-minD* construct, xylose concentrations in the range 0.05–0.3% were used. For induction of the P_*spac*_*-pgsA* construct, the final IPTG concentration was 100 μM. *B. subtilis* BFA2809 and its derivative IB1061 were grown in LB medium to mid-exponential phase in the presence of 100 μM IPTG. Subsequently, cultures were diluted 100-fold in media containing either FM 4-64 or FM 1-43 dyes in the presence or absence of 100 μM IPTG. Cells were shaken for a further 2 h, harvested by centrifugation, washed several times in dye-free medium and examined by fluorescence microscopy.

### Strains

The *B. subtilis* strains used here are derivatives of the wild-type strain PY79 (Youngman *et al*., 1984) and are listed in Table 1. Strain IB1056 in which *minD* is disrupted was constructed by integration of plasmid pSGminDdel into MO1099. Integration mutants were selected for chloramphenicol and erythromycin/lincomycin resistance.

In order to create strain IB1059, the plasmid pSGminD was introduced into IB1056 by ectopic integration at the *amyE* locus. Integration mutants were selected for spectinomycin and chloramphenicol resistance. To prepare strain IB1062, the plasmid pSGminD2 was introduced into MO1099 by ectopic integration at the *amyE* locus. Integration mutants were selected for spectinomycin resistance. The strains IB1060 and IB1061, expressing GFP–MinD, were prepared by introducing the plasmid pSGminD into MO1099 and BFA2809, respectively, by ectopic integration at the *amyE* locus with selection for spectinomycin resistance. GFP–MinD is a functional protein as judged by experiments which show that *gfp–minD* complements *minD* deletion mutant strains. The concentration of GFP–MinD in IB1060 grown in the presence of 0.05% xylose is expected to be similar to that of MinD in wild-type (PY79) cells as judged by Western blot analysis and the observation that cell growth and cell division were unaffected.

To create strains with GFP–MinD C-terminal deletions, plasmids pSGminDΔ3, pSGminDΔ4, pSGminDΔ6, pSGminDΔ15 and pSGminDΔ24, representing MinD truncations of 3, 4, 6, 15 and 24 amino acids, respectively, were introduced into IB1056 by integration at the *amyE* locus. Integration mutants were selected for spectinomycin and chloramphenicol.

### Plasmids

Plasmids (Table 1) were constructed using standard methods and amplified in *E. coli* MM294. All PCR fragments were amplified from PY79 chromosomal DNA. To construct pSGminDdel, a 469 bp DNA fragment of the *minD* gene (from 120 to 566 bp) was amplified using primers minDdelS (5′-GTAGATACTGATCTAGAACTGCGCAACCT-3′) and minDdelE (5′-CGATTTCGTCGAATTCCATCGTGTCAC-3′). The PCR fragment was digested with XbaI and EcoRI (sites are underlined in the primer sequences) and cloned into pSG1151 cut with the same restriction enzymes.

To construct pSGminD, which carries *gfpmut1* fused to the 5′ end of the *minD* coding sequence with expression directed by P_xyl_, a 875 bp DNA fragment containing the entire *minD* gene was amplified using the primers minDXhoIS (5′-CTAACAAGGCTTGAGGCTCGAGTGTGAATTGGG-3′) and minDEcoRIE (5′-GCATATGCTTTGTCGAATTCTTCTCTTTGATTC-3′). The PCR fragment was digested with XhoI and EcoRI and cloned into pSG1729 digested with XhoI and EcoRI. As for pSGminD, a pSGminD2 plasmid was constructed by cloning the same PCR fragment, containing the entire *minD* gene, into pSG1154.

To construct plasmids pSGminDΔ3, pSGminDΔ4, pSGminDΔ6, pSGminDΔ15 and pSGminDΔ24 which carry *gfpmut1* fused to the 5′ end of the *minD* coding sequence with different truncations from 3′ end and with expression controlled by P_xyl_, DNA fragments of *minD* gene were amplified using the sense primer minDXhoIS (5′-CTAACAAGGCTTGAGGCTCGAGTGTGAATTGGG-3′) and following antisense primers minDdel3 (5′-CTCTTTGATTCTATCACATTAGAATTCTCATCCGAAAAATG-3′), minDdel4 (5′-TGATTCTATCACATTAAGAGAATTCTCAGAAAAATGAC-3′), minDdel6/2 (5′-CACATTAAGATCTTACGAATTCTTATGACTTAATCTTAGCC-3′), minDdel15 (5′-TCTTAGCCATGAATTCTTAGTTTTGCTCTTCAAGC-3′), minDdel24/2 (5′-TGCTCTTCAAGCACGAATTCTCAAACAGATTCACCTAAG-3′). PCR fragments were digested with XhoI and EcoRI and cloned into pSG1729 digested with XhoI and EcoRI.

### Western blot analysis

GFP–MinD was quantified by immunoblot analysis. 20 ml cultures of cells grown in DSM supplemented with 0%, 0.05%, 0.1%, 0.3% and 0.5% xylose, respectively, were harvested and the cells re-suspended in 50 mM Tris-HCl pH 8.0, 1 mM EDTA, 100 mM NaCl, 1 mM AEBSF to an A_600_ equivalent to 20. The cells were lysed by sonication (3 × 20 s pulses). 20 μl of cell extract was mixed with 20 μl of 2× SDS-PAGE loading buffer and incubated in a boiling water bath for 10 min. Proteins were electrophoretically resolved on 12% (w/v) denaturing polyacrylamide gels (Laemmli, 1970) and transferred to Hybond-ECL membranes (Amersham Biosciences). The GFP–MinD fusion was detected using mouse anti-GFP antibodies (1:1000, Roche Diagnostics) and MinD was detected using mouse polyclonal anti-MinD antibodies (1:1000). Proteins were detected using a horseradish peroxidase-conjugated goat anti-mouse secondary antibody (1:5000, Promega) with an ECL kit (Amersham Biosciences).

### Fluorescence, confocal microscopy and image acquisition

*Bacillus subtilis* strains were grown as liquid cultures in LB or DSM (Harwood and Cutting, 1990). The cultures were inoculated from a fresh overnight plate to an OD_600_ of 0.1. For membrane visualization, the stains FM 4-64, FM 1-43 or FM 5-95 (Molecular Probes) were added at concentrations ranging from 0.2 up to 1 μg ml^−1^. NAO dye was used at concentrations ranging from 0.05 up to 0.1 μg ml^−1^. Cells were grown to mid-exponential phase, to an OD_600_ of 0.3–0.5. Cells were examined microscopically on freshly prepared 1.2% agarose-coated or poly l-lysine-treated slides for colocalization, FRAP, FRET or time-lapse fluorescence microscopy. Samples were also counterstained with DAPI (0.2 μg ml^−1^) to visualize DNA. When it was necessary to increase the cell density, cells were harvested by centrifugation (3 min at 10 000 r.p.m.) and re-suspended in a small volume of supernatant prior to examination by microscopy.

Immunofluorescence microscopy was carried out as follows. IB1062 vegetative cells in the mid-exponential stage of growth were fixed in 2.5% (v/v) paraformaldehyde, 0.008% (v/v) glutaraldehyde and 30 mM NaPO_4_ buffer pH 7.4 for 15 min at room temperature and then 30 min on ice. The fixed bacteria were washed three times in PBS (pH 7.4) and then re-suspended in GTE (50 mM glucose, 20 mM Tris-HCl pH 7.5, 10 mM EDTA) to a final A_600_ of approximately 0.2. Samples (10 μl) of fixed cells were distributed into wells of poly l-lysine-treated slides. Cell permeabilization was performed by lysozyme treatment (2 mg ml^−1^ lysozyme in GTE) for 3 min. After incubation with blocking agent (2% BSA in PBS) for 30 min, cells were incubated with a 1:500 dilution of mouse polyclonal anti-MinD antibody overnight at 4°C. Goat anti-mouse IgG conjugated to Alexa Fluor 488 (Molecular Probes) at a dilution 1:1000 was used for detection.

All images were acquired either with a Zeiss LSM 510 Meta confocal system with a Zeiss Axiovert 200 M inverted microscope or with an Olympus BX60 or BX61 microscopes, equipped with an Olympus DP30BW camera. The microscopic images were processed with LSM 5 image browser (Zeiss) or MicroImage software (Olympus). For image deconvolution and figure rendering, the Huygens Professional software package was used.

### Time-lapse microscopy and FRAP experiments

Time-lapse fluorescent microscopy and FRAP experiments were carried out with a Zeiss LSM 510 Meta confocal system with a Zeiss Axiovert inverted microscope, fitted with a Plan Apochromat 100×/1.4 oil objective and a temperature-controlled stage. The cells were prepared as described above. A 488 nm laser was used for excitation of GFP and emission collected through a 505–530 nm bandpass filter. Photobleaching was achieved by significantly increasing the laser power to the ROI. In these FRAP experiments, the strength of bleaching applied to the ROI was set at between 20 and 70 iterations. Typically, the number of iterations for photobleaching was the minimum needed to significantly photobleach the GFP fluorophore attached to MinD. A 543 nm laser was used for excitation of the FM 4-64 fluorophore. Again, photobleaching was achieved by significantly increasing the laser power applied to the ROI in the FRAP experiments. The length of bleaching was set at between 250 and 500 iterations within the ROI to ensure significant photobleaching of the FM 4-64 fluorophore attached to the lipid structures.

### FRET determination

In this work, acceptor-bleaching FRET was utilized. By using a fast scan speed and suboptimal resolution, we were able to record images rapidly which minimizing acquisitional bleach; however, this was at the expense of image quality. A ROI was drawn around selected FM 4-64 signals emanating from the lipid spiral structures. The area within this ROI was bleached using the 561 nm laser line. The acceptor fluorophore (FM 4-64) is difficult to fully photobleach with this laser line and, thus, the strength of bleach was set at between 250 and 500 iterations. Over this number of iterations, it was possible to bleach most of the signal from FM 4-64 within the ROI without any noticeable effect to the signal from GFP. A time series was also incorporated into the bleach script, and images were acquired every 2 s up to 3 min. Images were acquired using low, attenuated laser output to minimize acquisitional bleach. Bleaching of the FM 4-64 and any associated increase in GFP signal were monitored. The FRET efficiency (E), which is the percentage increase in GFP signal intensity after photobleaching of FM 4-64, was calculated according to Wouters *et al*. (1998). This determination of the FRET coefficient assumes that the majority, if not all, of the acceptor fluorophores (i.e. FM 4-64 in this case) are photobleached.

The mean FRET efficiency of all the measured ROIs was determined as 21 ± 6.2%. This FRET was further validated. One possibility was that the increase in GFP signal was due to the effect of the 561 nm laser line on the GFP fluorophore. IB1060 cells were therefore prepared as before, but without the FM 4-64 dye. A selected ROI associated with GFP–MinD spirals was bleached with the 561 nm laser. We observed an approximate 5% increase in the GFP signal. To further test this effect, we collected data on three separate occasions from more than 22 cells and more than 68 selected ROIs. The average ‘background’ FRET efficiency of all measured ROI was determined as 4%. This efficiency was subtracted from the previously determined value, giving a final value for the FRET efficiency of 17 ± 6.2%. In a further control experiment, the FM 4-64 fluorophore was bleached a second time to test the possibility of photorecovery of FM 4-64 which might have given rise to the measured FRET. In this case, E was essentially 0%, indicating that no recovery had occurred.
